# Quantitative assessment of interim PET in Hodgkin lymphoma: An evaluation of the qPET method in adult patients in the RAPID trial

**DOI:** 10.1371/journal.pone.0231027

**Published:** 2020-04-02

**Authors:** Thomas W. Georgi, Lars Kurch, Dirk Hasenclever, Victoria S. Warbey, Lucy Pike, John Radford, Osama Sabri, Regine Kluge, Sally F. Barrington

**Affiliations:** 1 Department of Nuclear Medicine, University of Leipzig, Leipzig, Germany; 2 Institute for Medical Informatics, Statistics and Epidemiology, University of Leipzig, Leipzig, Germany; 3 King's College London and Guy's & St Thomas' PET Centre, School of Biomedical Engineering and Imaging Sciences, Kings College London, London, United Kingdom; 4 University of Manchester and Christie National Health Service Foundation Trust, Manchester, United Kingdom; Spedali Civili of Brescia, University of Brescia, ITALY

## Abstract

**Aim:**

qPET is a quantitative method used to assess FDG-PET response in lymphoma. qPET was developed using 898 scans from children with Hodgkin Lymphoma (HL) in the EuroNet-PHL-C1 (C1) trial. The aim of this study was to determine if qPET could be applied as an alternative response method in adults in the RAPID trial.

**Methods:**

PET-CT scans performed after 3 cycles of ABVD in RAPID were re-evaluated by an independent reader, blinded to PET results and outcome in RAPID. All initially involved regions were assessed visually and by qPET. The distribution of qPET measurements was compared for RAPID and C1 patients. Previously published qPET thresholds corresponding to visual DS (vDS) of 1–5 in C1 were used to derive quantitative DS (qDS) for RAPID patients.

**Results:**

PET-CT scans were available for 450 patients from RAPID. vDS were 1 (171 scans), 2 (153 scans), 3 (72 scans), 4 (31 scans) and 5 (23 scans) respectively. The distribution of qPET values was similar to C1 patients, with a unimodal ‘normal’ distribution and a long tail to the right, suggestive of favorable response in the majority and less favorable response in the minority with outlying values. qPET thresholds from C1 applied in RAPID patients gave 86% concordance for vDS and qDS. There was 97% concordance for complete metabolic response (CMR; DS 1–3) vs. no-CMR using the Lugano classification.

**Conclusion:**

qPET which was developed in pediatric patients receiving more intensive OEPA chemotherapy, was a suitable quantitative method for assessing response in adult patients treated with ABVD in a response-adapted setting in the RAPID trial.

## Introduction

Achieving a balance between sufficient treatment to enable a high chance of cure whilst minimising toxicity to avoid late effects is a major concern in the treatment of Hodgkin lymphoma (HL) [1,2], especially for younger patients [3,4]. Recent studies have explored response-adapted treatment [5–7] including omission of radiotherapy in patients with a complete metabolic response (CMR) to chemotherapy [8–11]. Interim F-18-FDG-PET (iPET) has proved to be a reliable imaging tool for early treatment adaptation [12–16]. The current standard for iPET evaluation is the Lugano classification, which uses the Deauville score (DS), a five-point ordinal scale, to grade residual uptake, if present, in sites initially involved by lymphoma [17].

A purely visual iPET assessment using the DS may be prone to inaccuracies, with substantial inter reader variability [18–20]. Besides missing an area of uptake due to lymphoma (detection error), variability stems from a) trying to compare the degree of FDG uptake in small lymphoma residuals with uptake in normal reference organs (e.g. liver or mediastinum) separated by distance and b) because the visual impression of the degree of uptake is influenced by the adjacent background (simultaneous contrast illusion) [21]. The DS although graded, still results in uptake being ‘binned’ into five categories. A continuous scale might be preferable for assessing the risk of treatment failure for an individual patient than an ordinal scale [5].

The quantitative ‘qPET’ method was developed by members of our group to try and address some of these issues by introducing semi-automatic quantitative measurements. The method was derived from measurements made on iPET scans from 898 paediatric HL patients enrolled in the EuroNet-PHL-C1 (C1) trial [22,23] and is being used in the EuroNet-PHL-C2 (C2) trial [24] with currently more than 1700 patients registered. The qPET value is calculated as the ratio of the peak standardised uptake value (SUV) of the hottest residual ‘lymphoma’ uptake over the mean SUV in the liver. Using the mean liver uptake rather than the maximum liver uptake, which is currently suggested in international guidance for assigning DS 4 and 5, may be less influenced by noise in the image and yet still reproducible between observers [12].

The histogram of qPET values obtained from analysing scans from pediatric patients in the C1 trial showed a mixed distribution. The majority of qPET values had a normal distribution located to the left of the graph with the mode (most frequently occurring qPET value) at 0.95 with a long tail to the right which included the minority of outlying qPET values. This distribution is suggestive of a favorable metabolic response in the majority of patients with values in the normally distributed part of the histogram and a less favorable response in the minority with outlying values. Based on this assumption, qPET cut-off points of 1.30 and 2.00 would discriminate favorable from unfavorable response with high sensitivity or high specificity, respectively.

In order to translate qPET measurements into ‘quantitative’ Deauville scores (qDS), thresholds corresponding to visual Deauville scores (vDS) were also derived for the C1 patients. The lower qPET threshold values that corresponded to vDS of 3, 4 and 5 were 0.95, 1.30 and 2.00, respectively. It is noteworthy that the two thresholds in the histogram that distinguished patients with unfavorable response from those with favorable response with high sensitivity or high specificity respectively corresponded exactly to the qPET thresholds linking the visually and quantitatively assessed DS of 4 (above liver) and 5 (markedly above liver) [12,15]. This suggests that these qPET thresholds have biological relevance for lymphoma response assessment.

The iPET assessment in the ongoing C2 trial is performed using qPET. Scans from patients with a qPET value < 1.30 (i.e. qDS 1–3) are considered to represent iPET ‘negative’ results. Radiotherapy is omitted for these patients after chemotherapy. Up until now, the qPET method has only been evaluated in pediatric patients with HL after receiving two cycles of Vincristine, Etoposide, Prednisone and Adriamycin (OEPA) [25,26]. We hypothesised that a similar distribution of qPET values might be observed and that qPET thresholds derived from the C1 population might correspond to vDS in a population of adult patients treated with Adriamycin, Bleomycin, Vinblastine, Dacarbazine (ABVD) [27,28] in the RAPID trial [9]. This could allow this quantitative extension of the visual DS, qPET, to be applied as an alternative method to assess response in adult patients treated with ABVD.

## Methods

### Patients

602 patients were enrolled in the RAPID trial in the United Kingdom (UK) between 2003 and 2010 [9]. The RAPID trial included patients with stage IA or IIA classical HL, aged between 16 and 75 years. Patients with mediastinal bulk (maximal mediastinal diameter ≥ 33% of the internal thoracic diameter) were excluded. Patients received three cycles of chemotherapy with ABVD. Thereafter, a PET or PET-CT scan was performed at centres within the UK National Cancer Research Institute (NCRI) PET Network in 571/602 patients. Central review was performed by two experienced readers using a five-point scale which later evolved into the Deauville score. The 145 patients with a positive iPET scan result (score 3–5) received a fourth cycle of ABVD and involved field radiotherapy. 420 of the iPET negative patients (score 1–2) were randomized 1:1 to receive either radiotherapy (n = 209) or no further treatment (n = 211) [9].

This subsidiary analysis was not subject to a formal amendment. A subset of data was shared with care taken to render it practically anonymized to the recipient. Review of PET data was included in the original protocol that was approved as part of the ethics application. Personal details were removed from the scans prior to transfer to the central reviewers at the imaging core lab and labelled with a trial specific ID number and patient initials. All patients enrolled in the RAPID study gave written informed consent. Ethical approval was granted by the North West Multicentre Research Ethics Committee (MREC 03/8/056).

For the purpose of this analysis, the trial number and patient initials were removed and replaced by a new ID (from 1–602) which was a unique identifier not associated with the original study. The key to de-identify the patients is held by a separate organisation from the imaging core lab at University College London CRUK Cancer trials unit. Hence it was not possible for investigators in this analysis to identify patients.

All available iPET-CT scans from enrolled patients were re- evaluated by an independent reader (S1 Raw data). Patients with ‘PET only’ scans were excluded from the analysis. All iPET-CT scans were evaluated using Hybrid3D viewer software (HERMES Medical Solutions, Stockholm, Sweden).

### Visual assessment

The iPET re-evaluation was performed by a nuclear medicine physician experienced in reading PET-CT scans from patients with HL, blinded to the original scan results from the RAPID trial and from patient outcomes. The reader was from a different institution to the RAPID study investigators who had centrally reviewed scans for the trial.

Baseline PET scans were not performed in the RAPID trial, however the reader was supplied with the site(s) of disease given on case record forms, taken from the baseline CT scan reports for patients as part of the trial. Sites of initial involvement on CT were recorded by anatomical region as cervical, supraclavicular, axillary, mediastinal and hilar (right and/or left) and other regions (e.g. Waldeyers ring, submandibular, inguinal). The lesion with the highest residual uptake was used for the vDS as established in international guidelines [12].

### Quantitative assessment

**Fig 1:**
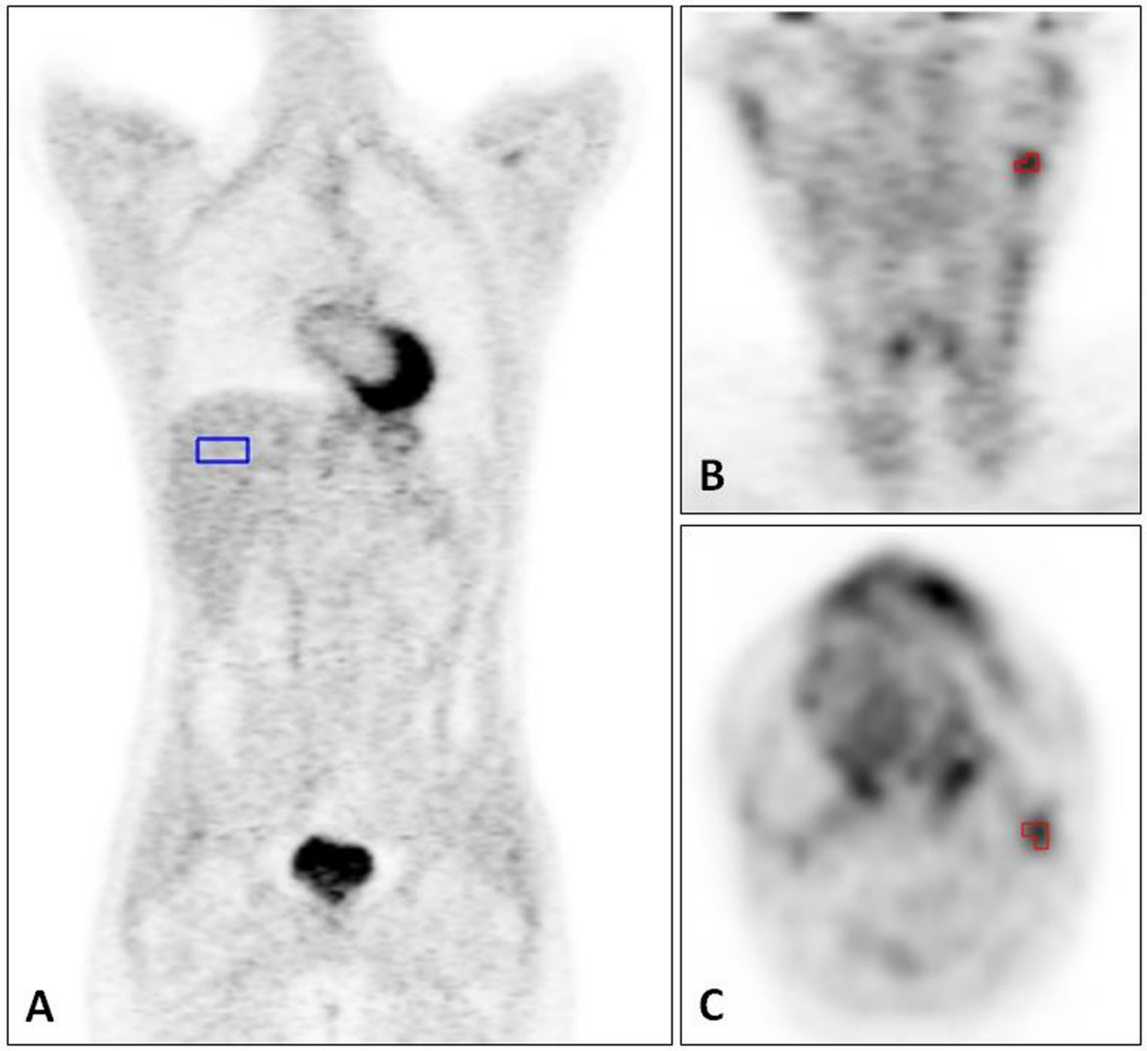
Interim-PET scan of a patient in the RAPID trial after three cycles of ABVD with qPET measurements. A) 30ml VOI (in blue) placed in the right liver lobe, measuring the mean SUV of the liver. B and C) The four hottest connected voxels (in red) inside the tumour, measuring the peak SUV of the tumour.

In addition to the visual re-evaluation, all lesions were assessed quantitatively using the qPET method [22]. The qPET value is defined as the quotient of the average SUV of the four hottest connected voxels inside the tumour (peak SUV) and the mean SUV in the right lobe of the liver measured using a 30ml VOI with length:width:height proportions of 2:2:1. The qPET value was recorded for the lesion with the highest residual uptake (Fig 1).

The qPET thresholds derived from the C1 study were used to assign a qDS (Table 1).

**Table 1:** Classification of the quantitative Deauville score according to the qPET value.

qDS	qPET value of lesion with highest residual uptake
1	0 (no uptake)
2	< 0.95
3	≥ 0.95 but < 1.30
4	≥ 1.30 but < 2.00
5	≥ 2.00

## Results

450/571 RAPID patients had iPET-CT scans that were available for visual re-evaluation. 445 scans were analysed using the qPET method. For two patients, quantification was not feasible due to prominent brown fat activity at sites where initial disease was reported on CT. In three patients, quantification was not possible because the data from an older Philips PET scanner was not stored in units of activity concentration and the Hybrid3D program was not able to convert the data to SUV values.

The assessment showed vDS of 1 (171 scans), 2 (153 scans), 3 (72 scans), 4 (31 scans) and 5 (23 scans) (Table 2).

**Table 2:** Comparison of visual and quantitative Deauville scores.

	qDS 1	qDS 2	qDS 3	qDS 4	qDS 5	n.e.*	sum
vDS 1	171	0	0	0	0	0	171
vDS 2	0	116	33	0	0	4	153
vDS 3	0	11	55	5	0	1	72
vDS 4	0	0	7	23	1	0	31
vDS 5	0	0	0	5	18	0	23
sum	171	127	95	33	19	5	450

*in five patients the quantification was not feasible (not evaluated).

**Fig 2:**
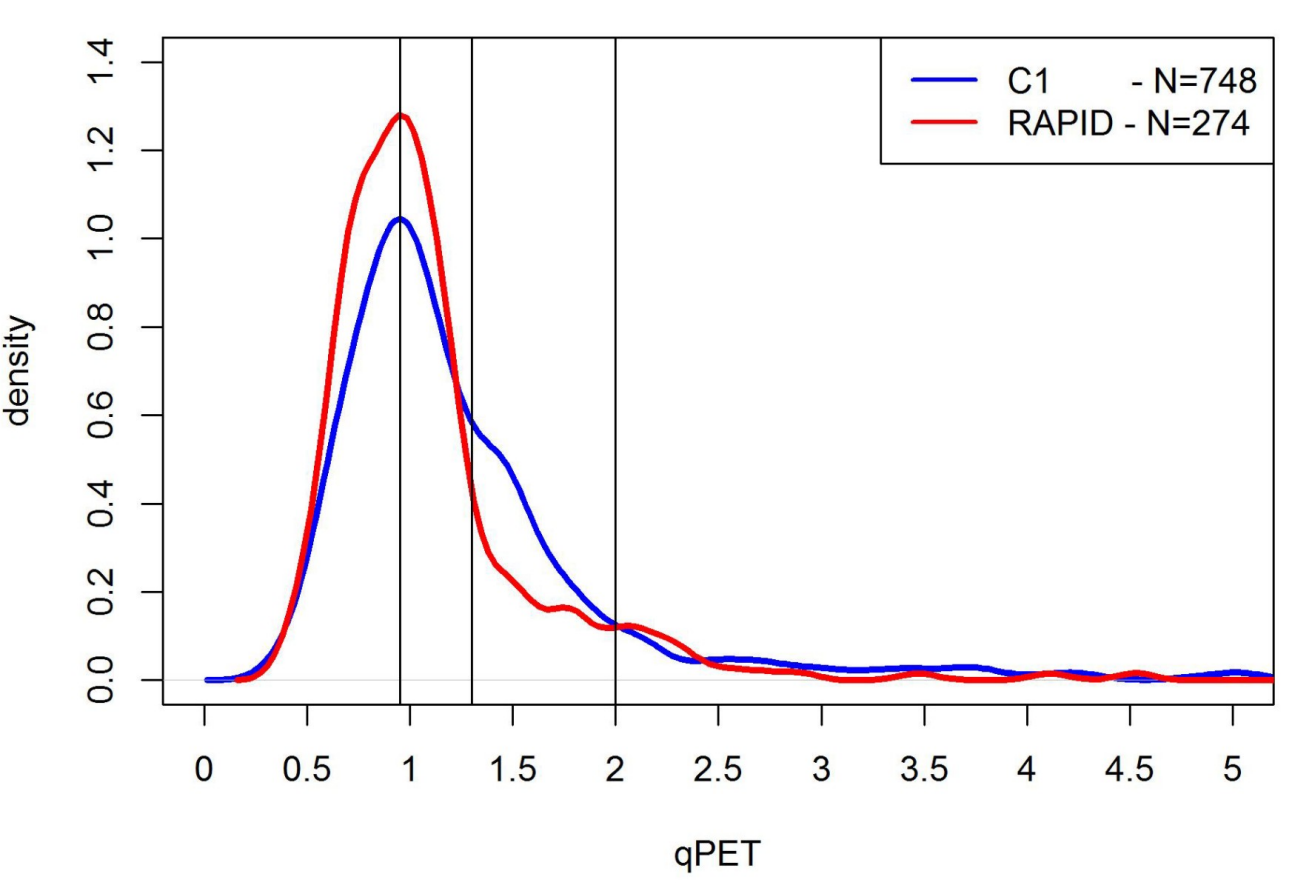
Density functions of the qPET measurements from the RAPID and EuroNet-PHL C1 trials. This figure shows the density function of the qPET measurements from the RAPID trial patient scans (in red) and the EuroNet-PHL C1 trial patient scans (in blue) as well as the qPET thresholds derived from the C1 study (black vertical lines; qPET ≥ 0.95 for qDS 3, qPET ≥ 1.30 for qDS 4 and qPET ≥ 2.00 for qDS 5). The functions appear to be similar, except the relative proportion of patients with qDS ≤ 3 was higher in the RAPID trial, which may be because the RAPID trial included only early stage patients. Hence, a higher proportion of patients with favourable response would be expected compared to C1.

**Fig 3:**
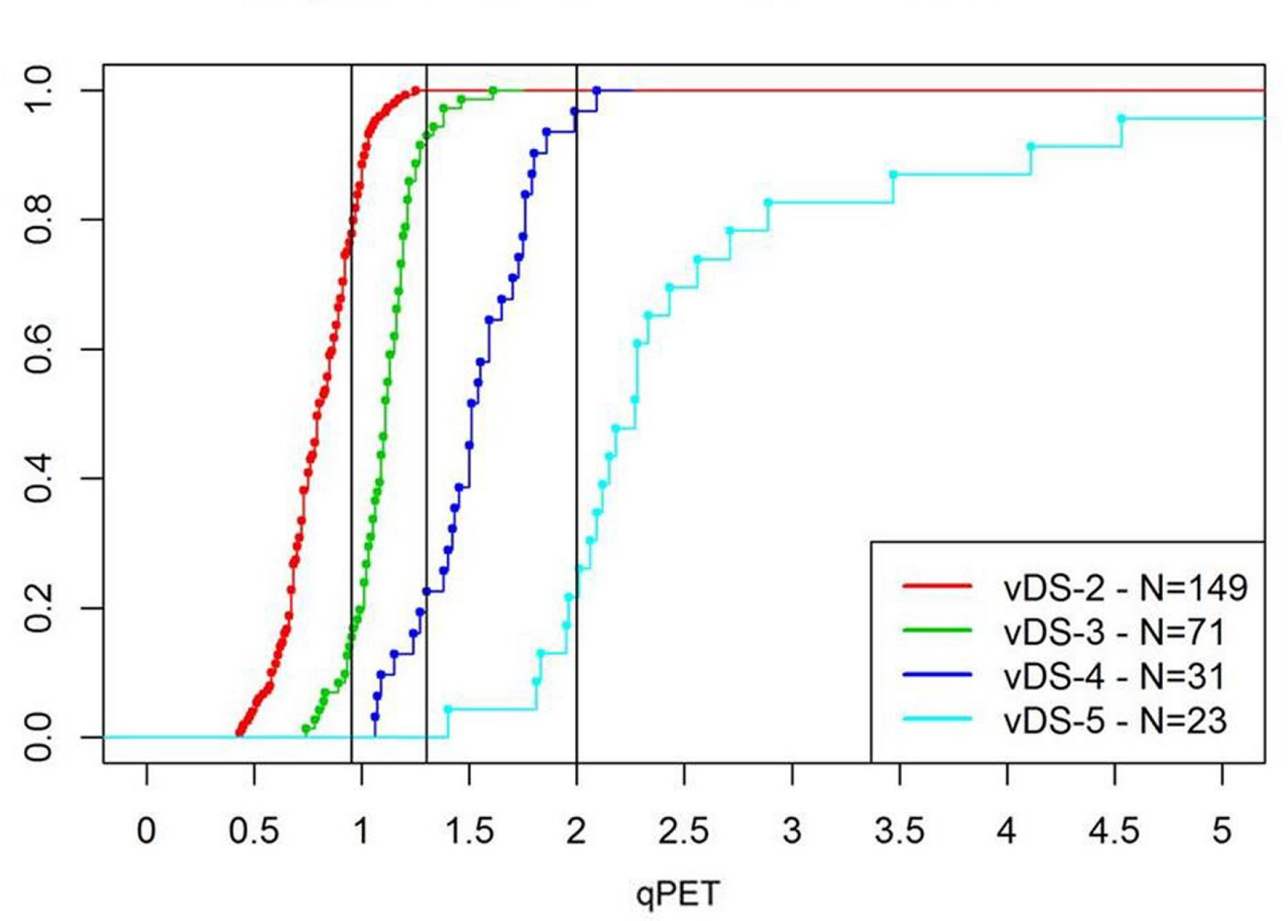
Empirical cumulative distribution functions of qPET in the RAPID trial. This figure shows the empirical cumulative distribution functions of the qPET values for patient scans with vDS of 2–5 in the RAPID trial. The black vertical lines represent the qPET thresholds for the qDS categories derived from the EuroNet-PHL-C1 study (qPET ≥ 0.95 for qDS 3, qPET ≥ 1.30 for qDS 4 and qPET ≥ 2.00 for qDS 5). These thresholds were also suitable for discriminating favourable from unfavourable response in the RAPID trial patients.

The qPET values showed a unimodal distribution (Fig 2), with a mode of 0.95 and a long tail to the right side similar to the mixed distribution previously reported in the paediatric population of C1 patients [22]. The qPET values for 274 patients with vDS of 2–5 were also plotted as empirical cumulative distribution functions (Fig 3). The 171 patients with a vDS of 1 had, by definition, no residual uptake and therefore the qPET value for these patient scans was zero. Using the qPET thresholds derived from pediatric patient scans in the C1 study of 0.95, 1.30 and 2.00, 127 RAPID patients had a qDS of 2, 95 patients a qDS of 3, 33 patients a qDS of 4 and 19 patients a qDS of 5, respectively (Table 2). The relative proportion of scans with qDS of 1–3 was higher in the RAPID re-analysis (88%) than in the C1 trial (72%) likely related to the different clinical characteristics of the two study populations.

Concordance between vDS and qDS was high at 86% (383/445) (Table 2). For the 62 cases where there was discordance, vDS and qDS differed by one score only. The greatest shift in scores was observed between DS 2 and DS 3. 33/153 patient scans with vDS 2 had qDS of 3, whilst 11/72 patient scans with vDS 3 had qDS of 2.

In the RAPID trial, the binary decision between iPET ‘negative’ and iPET ‘positive’ used to decide whether patients were eligible for randomisation was made between score 2 and score 3 (mediastinal threshold); scores 1 and 2 were regarded as CMR. With reference to this cut-off applied in RAPID, the concordance rate between vDS and qDS in this re-analysis of RAPID scan data was 90% (401/445). The specific agreement for a positive rating, which is the likelihood that one method (qDS or vDS) rated the response as score 3–5 when the other method was rated also as score 3–5 was 93%, while the specific agreement for a negative rating, which is the likelihood that one method rated the response as CMR when the other method was also CMR was 84%. In 44 patients, the qDS obtained using the qPET method would have changed the vDS result, from ‘negative’ to ‘positive’ in 33 patients and conversely from ‘positive’ to ‘negative’ in 11 patients.

The Lugano classification recommends to use DS of 1–3 to define CMR with standard therapy. With reference to the Lugano cut-off, the concordance rate between vDS and qDS for CMR versus no-CMR was 97% (433/445). The specific agreement for a positive rating was 98%, while the specific agreement for a negative rating was 89%. In 12 patients the qDS obtained using the qPET method would have changed the vDS result, from CMR to no-CMR in 7 patients and from no-CMR to CMR in 11 patients.

## Discussion

The qPET method was developed in pediatric patients enrolled in the C1 trial for whom treatment was adapted according to the result of an iPET scan, with radiotherapy omitted in good risk patients with a favorable metabolic response. These patients in the C1 trial received more intensive chemotherapy (OEPA) than is typically used in the treatment of adults with early-stage lymphoma, who usually receive ABVD chemotherapy [5]. The key message from this re-analysis of scan data from the RAPID trial is that the qPET method is a suitable alternative way to assess response in adult patients with early stage HL treated with ABVD, using thresholds derived in the C1 trial, to generate a qDS.

Potential advantages of this approach are that it may improve interobserver agreement, especially among less experienced PET readers, and provide a continuous scale. Specifically qPET, which is not influenced by simultaneous contrast illusion [21] nor involves comparisons of uptake over distances may reduce misinterpretation using a purely visual assessment. Compared to an ordinal scale, a continuous scale enables differentiated mathematical evaluation and may allow individual patient risk predictions if validated in future prospective trials. Cut-off points for favorable versus less favorable response could be optimised for specific treatment strategies and lymphoma subtypes.

The qPET distribution from patient scans in the C1 and the RAPID trials was very similar, with a pronounced peak around a qPET value of 0.95, suggesting these are the cases with favorable metabolic response with a tail of outlying values of cases with less favorable responses. The similarity in distribution suggests that the qPET distribution in HL may be independent of chemotherapy type, at least with respect to OEPA and ABVD. So far, the qPET method has not been evaluated in iPET scans from HL patients treated with other chemotherapy regimens such as Bleomycin, Etoposide, Adriamycin, Cyclophosphamide, Vincristine, Prednisone and Procarbazine (BEACOPP). Furthermore, it suggests that the qPET method may be applicable in adults with early stage disease as well as children undergoing PET response evaluation.

In the RAPID trial there was a higher proportion of patients with qDS of 1–3 than in the C1 trial. The RAPID trial included only patients with Ann Arbor stages IA and IIA without mediastinal bulk, with more patients experiencing a favorable metabolic response compared to the C1 trial.

The high concordance rate between vDS and qDS in RAPID using previously published qPET thresholds from C1 [22] to translate qPET measurements into qDS, suggests this is a suitable alternative way to assess response. It was not necessary to adapt the thresholds for adult ABVD treated patients. The 86% concordance rate between vDS and qDS is similar to that observed for visual assessment between two independent readers [18,29–31]. The greatest shift between visual and quantitative assessments was seen between DS 2 and DS 3 (the mediastinal blood pool reference region), which can be inferred mathematically from the fact that the quantitative threshold between qDS 2 and qDS 3 lies at the peak of the density values for patients with good metabolic response—meaning that there are many ‘borderline’ cases around this threshold. This has also been observed by other groups using visual assessment, with lower agreement even amongst expert readers for the mediastinal than the liver region [29,32].

We hypothesize that these thresholds may be transferable to some other lymphoma settings, since they likely depend more on the approximation of the visual impression of the degree of FDG uptake than on the tumour biology. The current clinically accepted definition of CMR [12,17,33] namely a vDS of 1–3 is supported by the form of the qPET distribution. Patients with a vDS of 1–3, approximately corresponding to qPET values below 1.3, represent patients with qPET values clearly within the “normal” peak.

The qPET measurement could be considered to be analogous to a laboratory parameter with a clearly defined normal range, as most HL patients have an excellent prognosis with a minority of cases with clearly abnormal results. This distribution depends on a large majority of patients showing a CMR and probably applies to HL but may be different for non-HL subtypes with poorer patient outcomes.

A limitation of both the qPET method and the DS is their sensitivity to differences in scan reconstructions and voxel sizes [5]. Efforts are currently being made to update harmonizing standards that will allow the impact of newer reconstruction methods on image interpretation to be assessed [34,35]. Standardisation of patient preparation, scan acquisition and reconstruction parameters is required but this applies to both visual and quantitative assessment of response [36]. Post filtering has been suggested as a way to standardise SUV values from different scanners, in particular when resolution or point spread function (PSF) modelling is used [37]. Currently no accepted standard for a quantitative method supporting the DS exists. A commonly used procedure is to compare the maximum SUV in tumour with the maximum uptake in the liver alongside visual assessment [5]. Recent data suggest that advanced image reconstructions (e.g. PSF, qCLEAR) over-estimate maximum SUV values compared to peak and mean SUV values [35]. Hence peak SUV has recently been proposed instead of maximum SUV for assessing tumour activity. The peak SUV was defined for this purpose as a sphere with a diameter of 12mm positioned to yield the highest activity [35] to approximate to the original definition as a 1ml spherical VOI. The peak SUV is less sensitive than the maximum SUV to noise and variation in imaging quality resulting from different reconstruction settings and could be more suited to more modern reconstructions [35]. The benefit of using peak rather than maximum SUV is however offset by the low recovery of counts in lesions smaller than 17mm which is common in iPET scans of HL patients, unlike solid cancers. Kaalep et al. [35] suggest that by reducing the VOI size, the peak SUV might become an effective alternative to maximum SUV, especially if quantitative comparison among reconstructions of non-harmonized PET/CT systems is desired. The qPET method fulfils these requirements.

In conclusion, this re-analysis of scans from patients in the RAPID trial suggests that qPET which was developed in paediatric patients receiving more intensive OEPA chemotherapy, is a suitable alternative method for assessing response in adult patients treated with ABVD in a response-adapted setting.

## Supporting information

S1 Raw Data qPET in RAPID trial patients raw data. (XLSX)
